# Mitochondrial genome characterization, evolution and intron dynamics of the entomopathogenic genus *Cordyceps*

**DOI:** 10.3389/fmicb.2025.1605218

**Published:** 2025-06-13

**Authors:** Jinlong Jia, Chengpeng Li, Mei Tang, Hefa Liao, Zhuliang Yang, Yanan Wang, Ming Li, Wenbo Zeng, Yuanbing Wang

**Affiliations:** ^1^Sanqi Medicines College, Wenshan University, Wenshan, China; ^2^College of Life Sciences, Yunnan University, Kunming, China; ^3^State Key Laboratory of Phytochemistry and Natural Medicines, Kunming Institute of Botany, Chinese Academy of Sciences, Kumming, China; ^4^Biotechnology and Germplasm Resources Institute, Yunnan Academy of Agricultural Sciences, Kunming, China; ^5^Key Laboratory for Plant Diversity and Biogeography of East Asia and Yunnan Key Laboratory for Fungal Diversity and Green Development, Kunming Institute of Botany, Chinese Academy of Sciences, Kunming, China; ^6^State Key Laboratory for Quality Assurance and Sustainable Use of Dao-di Herbs, Beijing, China; ^7^Yunnan International Joint Laboratory of Virology and Immunology, School of Life Sciences, Yunnan University, Kunming, China

**Keywords:** *Cordyceps*, mitochondrial genome, intron, phylogenetic analysis, comparative genomics

## Abstract

**Introduction:**

*Cordyceps* is a diverse genus of insect-pathogenic fungi, some of which have significant medicinal value.

**Methods:**

The complete mitogenomes of six *Cordyceps* species (*C. cicadae*, *C. cocoonihabita*, *C. militaris*, *C. neopruinosa*, *C. pruinosa*, and *C. tenuipes*) were sequenced, assembled, and annotated in this study. Additionally, the previously published mitogenome of *C. blackwelliae* was also included for comparative analysis.

**Results:**

The mitogenomes of these seven *Cordyceps* species are circular DNA molecules ranging length from 29,929 to 51,692bp, containing 15 protein-coding genes (PCGs), two rRNAs, and 25–27 tRNAs, as well as four to 21 open reading frames (ORFs). The *rps3* gene appears to be under relaxed selection pressure across *Cordyceps* species. The non-conserved PCGs, such as homing endonucleases and proteins of unknown functions, show dynamic evolutionary patterns, highlighting the genetic diversity among the species. Introns, recognized as key contributors to mitogenome size variation, frequently undergo gain and loss events, further contributing to genomic diversity. The comparative analysis revealed both conserved and variable elements within the mitogenomes of the seven *Cordyceps* species. Phylogenetic analysis using 14 PCGs generated a well-supported topology that clarified the evolutionary relationships among *Cordyceps* species.

**Discussion:**

In conclusion, this study provides valuable insights into the conservatism and variability of mitogenomes within the genus *Cordyceps*, enhancing our understanding of their origin, evolution, and genetic diversity.

## Introduction

*Cordyceps*, the most abundant taxon of entomopathogenic fungi in the family Cordycipitaceae, comprising approximately 180 known species.^1^ Species of this genus have a wide host range, infecting insects from diverse orders and spiders, including those isolated from soil and acting as plant endophytes (Fan et al., 2019). The fungi show remarkable geographic diversity, being mainly recorded from China, Japan, Thailand, Russia, Guatemala, Mexico, Norway, Denmark, France, Nepal, India, Mongolia, and Kazakhstan (Sung et al., 2007). Many cordycipitoid fungi have long been valued in traditional Chinese medicine and used as health supplements due to their diverse pharmacological properties, such as antibacterial, antiviral, and antitumor activities. The species *Cordyceps militaris* and *C. cicadae* (syn. Isaria cicadae) are well-known for their ability to enhance the human immune system (Yue et al., 2013; Zhang J. et al., 2015; Zhang Y. et al., 2015). In general, *Cordyceps* and related genera have beeen identified using molecular data derived from nuclear genes, including the small subunit ribosomal RNA (nrSSU), large subunit ribosomal RNA (*nrLSU*), translation elongation factor 1-alpha (*tef1*), RNA polymerase II subunit 1 (*rpb1*), and subunit 2 (*rpb2*) (Sung et al., 2007; Mongkolsamrit et al., 2020; Kepler et al., 2017). Although these nuclear gene have been extensively used to confirm the phylogenetic relationships among *Cordyceps* species, many species cannot be accurately identified (Kepler et al., 2020). The development of more comprehensive genetic analysis tools is necessary to facilitate a thorough phylogenetic investigation of *Cordyceps*.

Mitochondria and plastids, since their endosymbiotic origin, have undergone a complex process of nuclear integration, transitioning from independent bacteria to semi-autonomous organelles (Adams et al., 2002; Timmis et al., 2004; Kleine et al., 2009). These organelles are essential for numerous vital cellular processes, including cellular respiration, photosynthesis, lipid metabolism, as well as the synthesis of nucleotides and amino acids (Millar et al., 2005; van Wijk and Baginsky, 2011; Forsythe et al., 2019). Mitochondria are unique among organelles due to they have their own DNA, referred to as mitochondrial DNA (mtDNA) or the mitochondrial genome (mitogenome) (Burger et al., 2003). The mitogenome of fungi typically consists of 15 protein-coding genes (PCGs), which encode three ATP synthase subunits, seven NADH dehydrogenase subunits, one complex III (cytochrome c reductase), three complex IV (cytochrome c oxidase), and one ribosomal protein S3 (*rps3*) (Johnston and Williams, 2016). In contrast to nuclear genomes, mtDNA is characterized by the conservation of orthologous genes, uniparental inheritance, and multiple copies per cell (Sandor et al., 2018). These features make mtDNA a valuable molecular marker in eukaryotic ecology, phylogenetics, and population genetics (Corradi and Bonen, 2012; Galtier et al., 2009).

The number of fully sequenced fungal mitogenomes have significantly increased in recent years, thanks to advancements in sequencing technologies and the establishment of the Fungal Mitochondrial Genome Project (FMGP) (Husami et al., 2020; Paquin et al., 1997). To date, 28 complete mitogenomes from *Cordyceps* have been cataloged in the public database NCBI,^2^ including *C. tenuipes*, *C. militaris*, *C. pruinosa*, *C. cicadae*, and *C. blackwelliae*. These mitogenomes provide comprehensive data on diverse genomic features, including gene arrangement, intron dynamics, repeat sequences, and the expansion or contraction of intergenic regions (Li et al., 2020a). The non-conserved mitochondrial genes provides valuable insights into the origin and evolution of fungi (Wang X. et al., 2020; Zhang et al., 2017). The study of *Cordyceps* mitogenomes can enhance our understanding of the phylogeny and evolutionary mechanisms underlying entomopathogenic fungi. Despite these advancements, intraspecific and interspecific variations in the mitogenomes of *Cordyceps* remain insufficiently investigated. This gap limits our comprehensive understanding of the phylogeny and adaptive evolution within the family Cordycipitaceae.

In this study, the complete mitogenomes of *C. cicadae*, *C. cocoonihabita*, *C. militaris*, *C. neopruinosa*, *C. pruinosa*, and *C. tenupes* were sequenced, assembled, and annotated. Additionally, we included a comparison with the previously published complete mitogenome of *C. blackwelliae* (NC068268). Our objective encompassed three main aspects: firstly, we characterized the mitogenomes of these seven *Cordyceps* species; secondly, through a comprehensive comparative analysis, we discerned both commonalities and disparities among the mitogenomes; lastly, we conducted a extnesive molecular phylogenetic analysis of the order Hypocreales. The present study provides further knowledge about genomic evolution, phylogeny and genetic diversity of the significant entomopathogenic genus *Cordyceps*.

## Materials and methods

### Sample collection and sequencing

Six *Cordyceps* species were collected from various locations in Yunnan, China. Detailed information regarding their growth environments and collection sites was provided in Supplementary Table S9. Species were identified based on morphological observation and phylogenetic analysis of Internal Transcribed Spacer (*ITS*) sequences. The following specimens of *Cordyceps* species were examined: *C. cicadae* (KUNCC 6001), *C. cocoonihabita* (KUNCC 8672), *C. militaris* (KUNCC 7809), *C. neopruinosa* (KUNCC 8759), *C. pruinosa* (KUNCC 8674), and *C. tenuipes* (KUNCC 6172). All specimens are preserved at the Kunming Institute of Botany, Chinese Academy of Sciences (Contact information: YuanBing Wang, wangyuanbing@mail.kib.ac.cn). Genomic DNA was extracted from the six *Cordyceps* species using the Fungal DNA Extraction Kit (Sangon Biotech, Shanghai, China), following the manufacturer’s instructions.

### Mitogenomes assembly and annotation

Whole-genome sequencing (WGS) of the six *Cordyceps* species was performed using the Illumina XTen platform (Illumina Inc., San Diego, CA, United States). The mitogenomes were assembled using Getorganelle (v1.7.7.0) (Jin et al., 2020) and NOVOPlasty (v4.3.1) (Dierckxsens et al., 2017), resulting in completely assembled mitogenomes. Genome annotation was conducted using the online tools MFannot (Valach et al., 2014) and MITOS2 (Bernt et al., 2013), with mitochondrial genetic code 4. tRNAs and their secondary structures were identified using the tRNAscan-SE (v1.23) (Lowe and Chan, 2016) program. Finally, graphical representations of the mitogenomes were generated using the OGDraw (v1.3.1) (Lohse et al., 2007) online plotting tool.

### Sequence feature analysis

The nucleotide composition and codon usage of the seven *Cordyceps* mitogenomes were analyzed using Phylosuite (v1.2.3) (Zhang et al., 2020), applying genetic code “4.” Nucleotide composition skews were calculated using the following formulas: AT-shew = (A-T)/(A + T) and GC-shew = (G-C)/(G + C) (Perna and Kocher, 1995). Genetic distances between the 14 core PCGs (*atp6*, *atp8*, *atp9*, *cob*, *cox1*, *cox2*, *cox3*, *nad1*, *nad2*, n*a*d3, *nad4*, *nad4L*, *nad5*, and *nad6*) were calculated based on the Kimura-2 parameter (K2P) substitution model in MEGA7 (Caspermeyer, 2016). Non-synonymous (Ka) and synonymous (Ks) substitution rates for the 14 core PCGs of the seven mitogenomes were calculated using KaKs_calculator (v3.0) (Zhang, 2022). Genomic synteny between the seven mitogenomes was analyzed using BLAST and MCscanX (Wang et al., 2012).

### Repeat element analysis

Self-searches of the seven *Cordyceps* mitogenomes were conducted using BlastN with an *E*-value threshold of <10–10. Tandem repeat sequences were identified using Tandem Repeats Finder (v4.09) (Benson, 1999) with default parameters. Interspersed repeats were detected using REPuter (v2.1) (Kurtz, 2001), applying a Hamming distance of 3, a maximum repeat size of 5,000, and a minimum repeat size of 30. Simple sequence repeats (SSRs) were identified using MISA (v1.0) (Beier et al., 2017) under the following conditions: mononucleotide repeats 10, dinucleotide repeats 5, trinucleotide repeats 4, as well as tetranucleotide, pentanucleotide, and hexanucleotide repeats 3.

### Intron analysis

The majority of eukaryotic mitogenomes are typically intron less, fungi belonging to the order Hypocreales, including *Cordyceps*, frequently contain varying numbers of introns (Chen et al., 2021; Martin et al., 2007; Zhang J. et al., 2015; Zhang Y. et al., 2015). Following established methods (Cheng et al., 2021), the introns within the PCGs of *Cordyceps* mitgenomes were classified into Pcls using the reference genome of *Tolypocladium inflatum* (NC036382) (Zhang and Zhang, 2019). Initially, the PCGs from *Cordyceps* mitogenomes were aligned with the corresponding PCGs of *T. inflatum* using MAFFT (Férandon et al., 2010). Each Pcl represents introns inserted at the same position within the coding region of a given PCG (Cheng et al., 2021). Introns within the same Pcls exhibited high sequence similarity were considered orthologous (Férandon et al., 2010). Conversely, different Pcls generally exhibit low sequence similarity and often contain non-orthologous mobile genetic elements (Huang et al., 2021). The Pcls for the PCGs in *Cordyceps* mitogenomes were designated according to their insertion positions within coding regions of the reference host genes.

### Phylogenetic analysis

In this study, the phylogenetic tree for the order Hypocreales was constructed using maximum likelihood (ML) and Bayesian inference (BI) methods based on 14 core PCGs (*atp6*, *atp8*, *atp9*, *cytb*, *cox1*, *cox2*, *cox3*, *nad1*, *nad2*, *nad3*, *nad4*, *nad4L*, *nad5*, *nad6*). *Neurospora crassa* and P*odospora anserina* were used as outgroup taxa. The 14 PCGs were extracted using Phylosuite and subsequently aligned using MAFFT (v7.520) (Katoh et al., 2019). The resulting aligned sequences were then concatenated using SequenceMatrix (v1.7.8) (Vaidya et al., 2011). The ModelFinder (v1.6.12) (Kalyaanamoorthy et al., 2017) was employed to determine the optimal evolutionary model for the concatenated alignments. The ML analysis was performed using IQ-tree (v1.6.8) (Nguyen et al., 2015), with 1,000 ultrafast bootstrap replications under the edge-linked partition model, based on the Bayesian information criterion (BIC). The BI analysis was conducted using MrBayes (v3.2.7a) (Ronquist et al., 2012), where two independent runs were performed, each with four chains (three heated, one cold), running for 2 × 106 generations. Samples were taken every 100 generations. The convergence was assumed when the estimated sample size (ESS) exceeded 100 and the potential scale reduction factor (PSRF) approached 1.0. The first 25% of samples were discarded as burn-in, and the remaining trees were used to calculate Bayesian posterior probabilities (BPP) in the 50% majority-rule consensus tree (Li et al., 2021). The phylogenetic tree was visualized and edited using Figtree (v1.4.4).

## Results

### Mitogenome characterization of seven *Cordyceps* species

**Figure 1:**
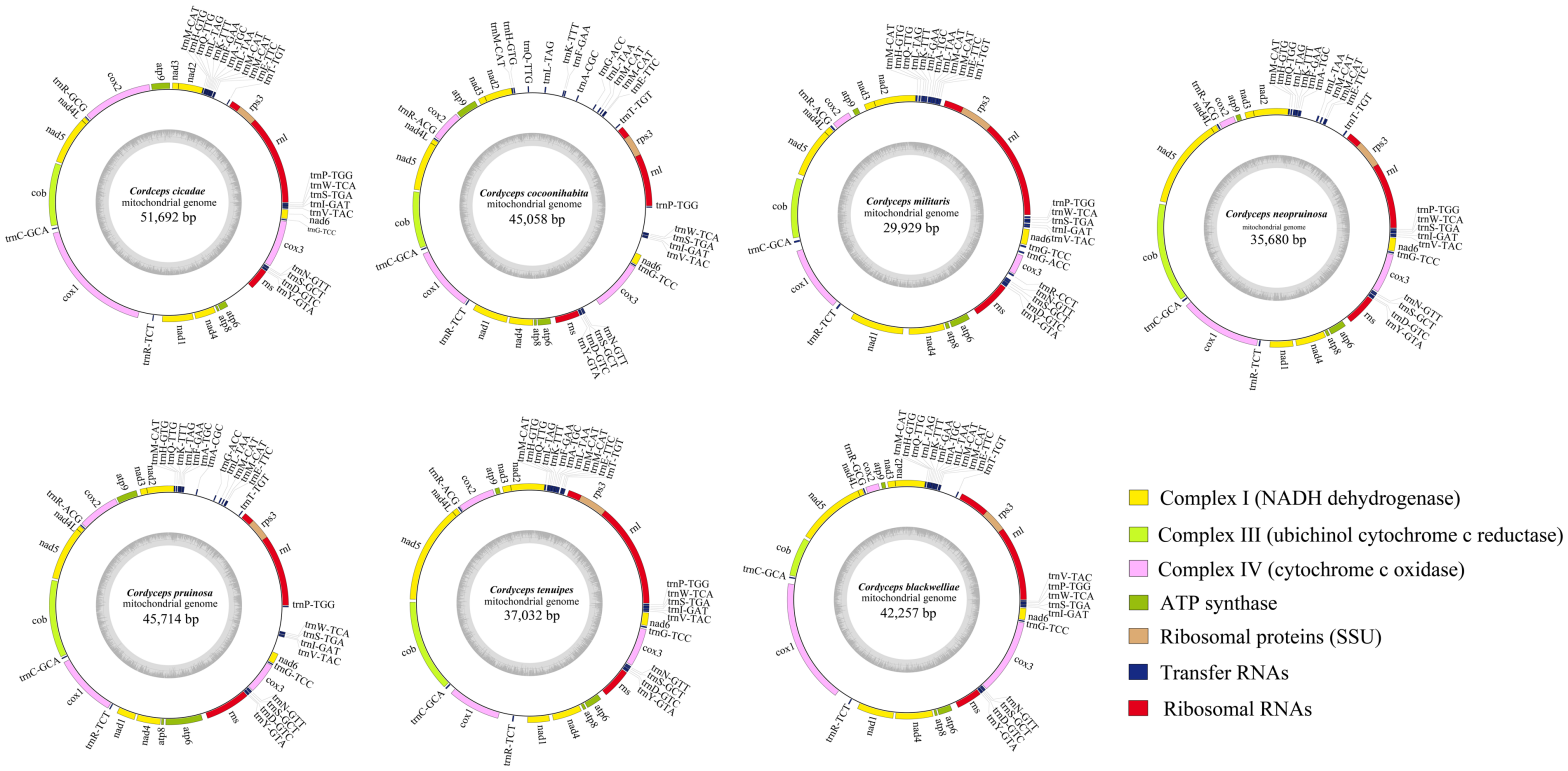
Circular maps of the mitogenomes of seven *Cordyceps* species. The genes with distinct functions are represented by different colored blocks. Genes located on the negative strand are shown inside the circle, while genes on the positive strand are displayed outside the circle.

The complete mitogenomes of the seven *Cordyceps* species were found to consist of single circular DNA molecules, with genome sizes ranging from 29,929 to 51,692 bp (Figure 1). Among these, *C. cicadae* had the largest mitogenome, while *C. militaris* had the smallest (Supplementary Table S1). The GC contents of the seven mitogenomes ranged from 25.1 to 26.7%, with an average GC content of 26.01%. The AT-skew of the *C. militaris* is negative, while the remaining species exhibited positive AT-skew values. All seven mitogenomes had positive GC-skew values. The number of ORFs ranged from four to 21, with *C. militaris* having the lowest count at four ORFs. These ORFs primarily encoded LAGLIDADG and GIY-YIG homing endonucleases, as well as proteins of unknown function. Additionally, a total of 84 introns were detected across the seven mitogenomes, with each species containing five to 21 introns, some of which included zero to two intronic ORFs. These introns were classified into five groups: 42 introns in group IB, 17 to group IA, eight in group ID, 12 in group IC1, and five in group IC2 (Supplementary Table S2). The seven species all shared a consistent set of genes, encompassing two ribosomal RNA genes (*rns* and *rnl*), 15 PCGs (*atp6*, *atp8*, *atp9*, cy*t*b, *cox1*, *cox2*, *cox3*, *nad1*, *nad2*, *nad3*, *nad4*, *nad4L*, *nad5*, *nad6*, and *rps3*), and 25 to 27 transfer RNA (tRNA) genes. The number of tRNA genes in these species varied, with three species having 25 genes, *C. cocoonihabita* having 26 genes, and *C. militaris* and *C. pruinosa* having 27 genes. The tRNA genes encoded the 20 standard amino acids, with lengths ranging from 71 to 85 bp, each exhibiting the typical cloverleaf structure (Supplementary Figure S1).

Mitogenomes of the seven *Cordyceps* species showed significant enlargement, prompting a detailed analysis of the composition of each gene region within the mitogenomes. The results indicated that while protein-coding regions were highly conserved across all species, there were substantial differences in introns and intergenic regions. Specifically, *C. cicadae* had a significantly higher number of nucleotides in its intronic regions compared to other species. In contrast, *C. cocoonihabita* had a markedly greater number of nucleotides in its intergenic regions compared to the rest of the species (Supplementary Figure S2).

### Codon usage analysis

To further investigate the conservation and variability within *Cordyceps* mitogenomes, we analyzed the codon usage across the seven species for the 15 PCGs. The majority of these PCGs employed AT* as the start codon. Specifically, ATG was used to initiate the genes *atp8*, *atp9*, *cox2*, *cox3*, *cytb*, *nad2*, *nad3*, *nad4L*, *nad5*, and *nad6*. In contrast, alternative start codons such as ATA, ATT, and TTA were employed by certain mitochondrial genes. Regarding stop codons in core PCGs of *Cordyceps* mitogenomes, TAA was predominantly used. However, the *nad3* and *rps3* genes ended with TAG in *C. tenuipes* (Supplementary Table S3).

**Figure 2:**
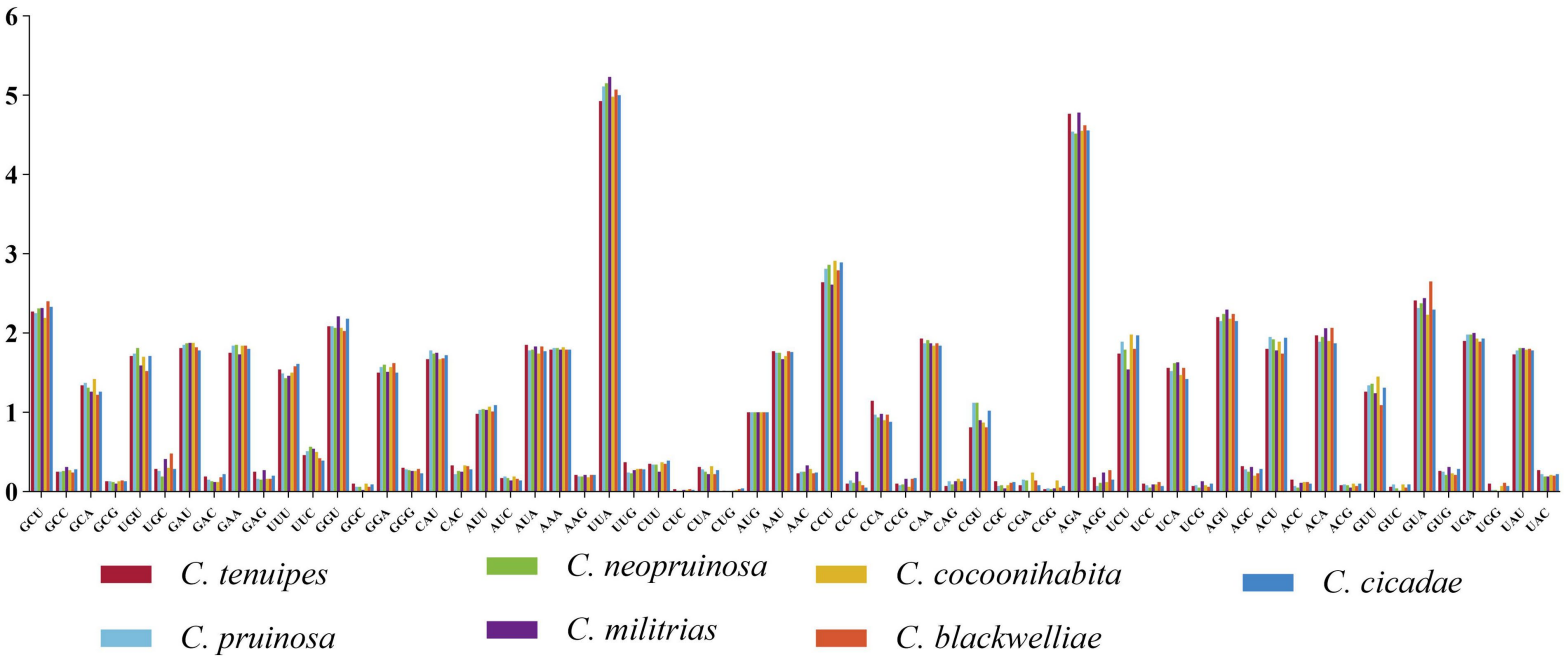
Codon usage analysis of the mitogenomes of seven *Cordyceps* species. The x-axis represents the codons, and the y-axis denotes the frequency of codon usage.

Codon usage analysis revealed that the codon preferences were highly similar in the seven *Cordyceps* mitogenomes. Among the 15 PCGs, UUA was the most frequently used codon, followed by commonly used codons such as UUU (for Phenylalanine; Phe), AUU (for Isoleucine; Ile), AUA (for Isoleucine; Ile), AAA (for Lysine; Lys), and UUA (for Leucine; Leu). The predominance of these codons contributed to the high AT content (74%) observed in the mitogenomes, as they frequently ended with A or T (Figure 2). The tRNA molecules in size ranged from 71 to 85 bp. Notably, the tRNAs for *trnL*, *trnS*, and *trnY* were longer than 80 bp and had additional arms, which significantly contributed to the length. Moreover, a considerable number of G-U mismatches were detected within the secondary structures of the tRNAs (Supplementary Figure S2).

### Repetitive sequence analysis

**Figure 3:**
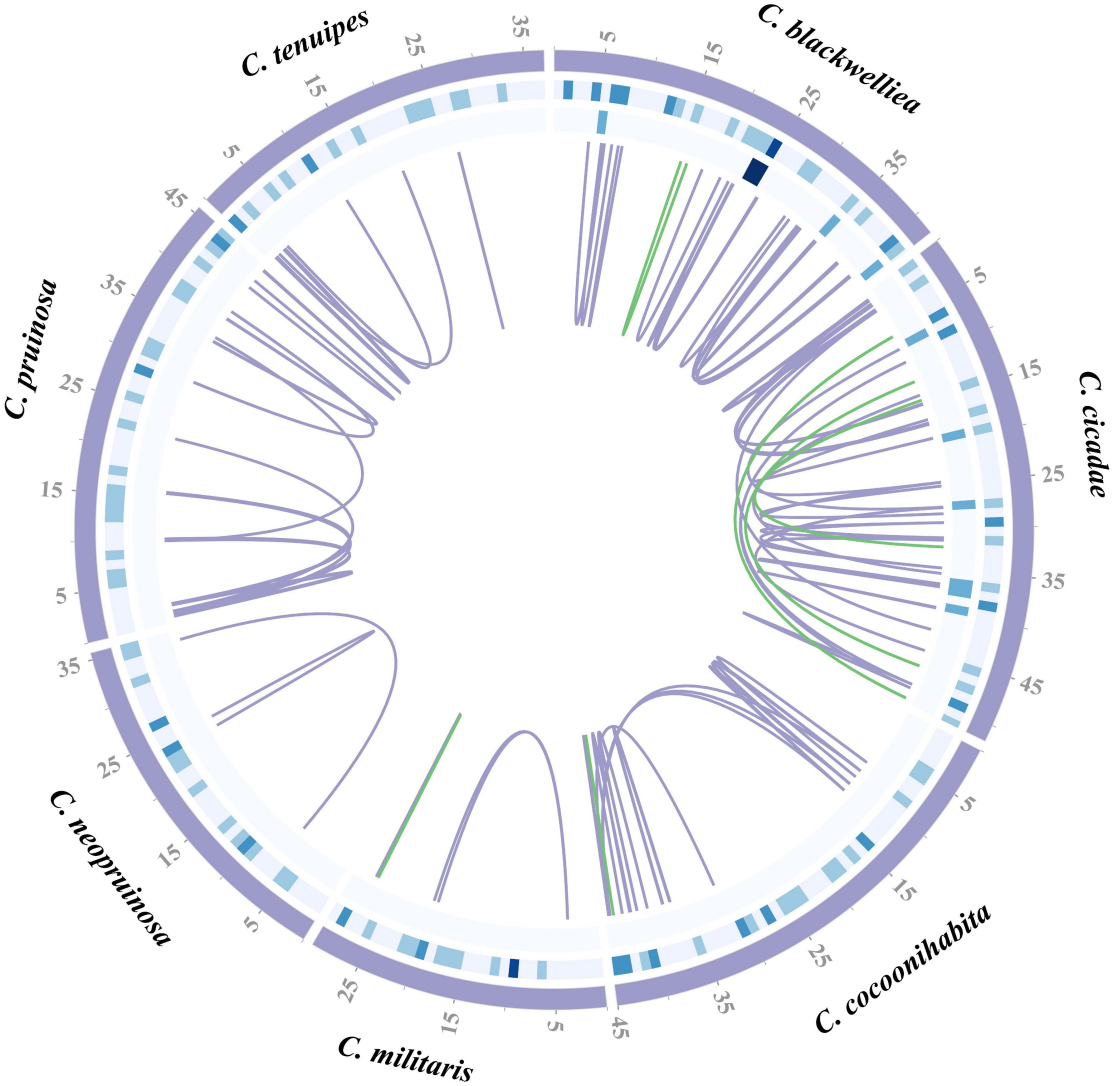
Distribution of repeat sequences in the mitogenomes of seven *Cordyceps* species. The circles in the diagram present the positions of dispersed repeats, tandem repeats, and simple repeats from innermost to outermost. The blue-gray lines indicate as forward repeats, while green lines represent palindromic repeats.

Repetitive sequences in the mitogenomes of seven *Cordyceps* species were identified using self-BLAST search. Numbers of repetitive regions detected in each species were as follows: ten in *C. blackwelliae*, 18 in *C. cicadae*, 17 in *C. cocoonihabita*, two in *C. militaris*, two in *C. neopruinosa*, 15 in *C. pruinosa*, and seven in *C. tenuipes*. These repetitive sequences ranged from 40 to 420 bp in length, with the longest occurring in *C. cocoonihabita* and the shortest in *C. blackwelliae* (Supplementary Table S4). Tandem repeat sequences were identified, with five in *C. blackwelliae*, six in *C. cicadae*, four in *C. cocoonihabita*, four in *C. militaris*, five in *C. neopruinosa*, four in *C. pruinosa*, and four in *C. tenuipes*. These tandem repeats with length ranged from six to 45 bp and had copy numbers ranging from 1.9 to 6 (Supplementary Table S5). The analysis detected various repeat sequences, including mononucleotide, dinucleotide, trinucleotide, tetranucleotide, pentanucleotide, and hexanucleotide repeats. Among these repeats, trinucleotide and tetranucleotide were found to be the most abundant. A total of 26 SSRs were identified in the mitogenome of *C. blackwelliae*, while *C. cicadae*, *C. cocoonihabita*, *C. militaris*, *C. neopruinosa*, *C. pruinosa*, and *C. tenuipes* had 21, 24, 15, 17, 21, and 15 SSRs, respectively (Supplementary Table S6). Furthermore, both forward (F) and palindromic (P) repeat sequences were found with a higher prevalence of forward repeats in the seven species (Supplementary Table S7). In terms of the distribution of repetitive sequences of the seven mitogenomes, the *C. cicadae*, with the largest mitogenome, had the highest number of repetitive sequences primarily located in introns and intergenic regions (Figure 3).

### Intron dynamics analysis

The number of introns showed a significant correlation with the sizes of the seven *Cordyceps* mitogenomes (*p* < 0.01). Pearson and Spearman correlation coefficients were 0.93 and 0.89, respectively, indicating a significant contribution of introns to the variation in *Cordyceps* mitogenomic size (Supplementary Figure S3). A total of 84 introns were detected across the seven mitogenomes, with each species harboring 5–21 introns. These introns also included zero to two intron ORFs, primarily encoding LAGLIDADG homing endonucleases, GIY-YIG homing endonucleases, and proteins with unknown functions. The study indicated that intron loss or gain events occurred throughout the evolutionary history of *Cordyceps*. Among these introns, 76.19% (64 introns) were located within core PCGs, while 23.81% reside within rRNA genes, making core PCGs the primary repository for intron. Introns were distributed in the *atp9*, *cox2*, *nad5*, *cob*, *cox1*, *atp6*, *nad1*, *nad4*, and *cox3* genes, however, introns lack in the *atp6*, *nad2*, *nad3*, *nad4L*, *nad6*, and *rps3* genes (Supplementary Figure S3). This uneven distribution of introns suggested a gene preference, with the majority of introns targeting core PCGs.

**Figure 4:**
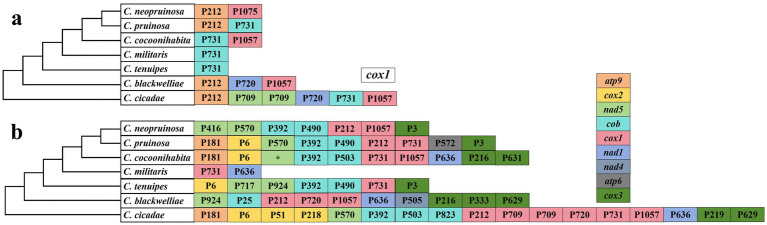
Positional classification (Pcl) of introns within *cox1* **(a)** and other core PCGs **(b)** of seven *Cordyceps* species. Pcls, representing directly homologous introns, are labeled based on their insertion site (nucleotide positions) relative to the reference gene (*cox1*, GenBank accession: NC036382). The phylogenetic relationships among the seven *Cordyceps* species were inferred using Bayesian Inference (BI) and Maximum Likelihood (ML) methods, derived from a combined set of PCGs.* represented undetermined positional classification.

Based on the insertion sites of introns within the protein-coding regions in the host genes, the introns were classified into different positional categories (Pcls) corresponding to their reference genes. The *cox1* and *cob* genes contained the highest number of introns, with 17 and 14, respectively, exhibiting the greatest variation in intron count. The introns in the *cox1* gene of seven *Cordyceps* species were categorized into P212, P709, P720, P731, and P1057, with P212, P731, and P1057, being the most widely distributed. Among these species, *C. cicadae* contained all intron types, whereas *C. militaris* and *C. tenuipes* showed significant intron loss in the *cox1* gene, retaining only the P731 intron (Figure 4a). The *atp6*, *atp9*, *cox2*, *cox3*, *nad1*, *nad4*, and *nad5* genes exhibited the presence of 1, 1, 3, 6, 2, 1, and 5 Pcls, respectively. The *cob* gene P392 contained the most abundant intron P392 across the *Cordyceps* species, while P6 from the *cox2* gene was widely distributed in *C. cicadae*, *C. cocoonihabita*, *C. pruinosa*, and *C. tenuipes*. Conversely, some introns, such as P572 from *atp6*, P25 and P823 from *cob*, P51 and P218 from *cox2*, P333, P219, and P631 from *cox3*, P505 from *nad4*, as well as P717 and P416 from *nad5* were detected within a single species, as rare Pcls in Cordyceps (Figure 4b). Interestingly, these rare Pcls were also found in distantly related species such as *Ganoderma lingzhii* (Li et al., 2022) and *Agaricus bisporus* (Férandon et al., 2010). These findings suggest the possibility of intron transfer within the mitochondrial genome of *Cordyceps*, and highlight the convergent nature of intron insertion across distantly related species.

### Analysis of variability, genetic distance, and evolutionary rate of PCGs

**Figure 5:**
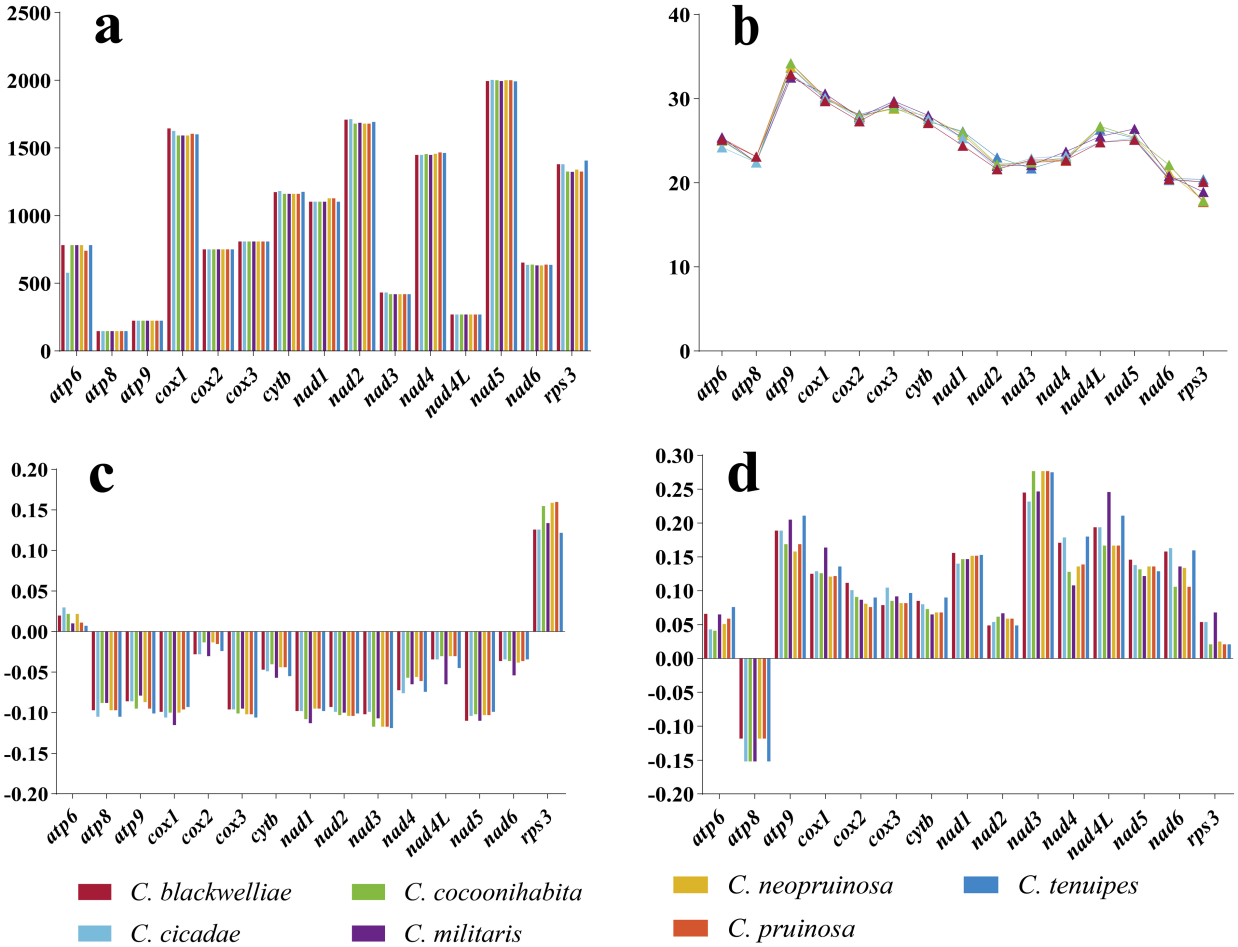
Variation in length and base composition of the 15 PCGs in the mitogenomes of seven *Cordyceps* species. **(a)** Variation in the length of 15 PCGs; **(b)** Variation in GC content; **(c)** Variation in AT-skew; **(d)** Variation in GC-skew.

The lengths of the *atp8*, *atp9*, *cox2*, *cox3*, and *nad4L* genes were identical in the mitogenomes of the seven *Cordyceps* species. Notably, the *cox1* gene exhibited the most prominent variation among these species. Among the 15 PCGs, the *atp9* gene exhibited the highest GC content, ranging from 32.5 to 34.2%, with an average of 33.46%. In contrast, the *rps3* gene had the lowest GC content, ranging from 17.7 to 20.4%, with an average of 18.99%. The *atp6* and *rps3* genes exhibited positive AT-skew values, while the remaining 13 genes exhibited negative values. The *atp8* gene had a negative GC-skew value whereas all other 14 genes had positive values (Figure 5).

**Figure 6:**
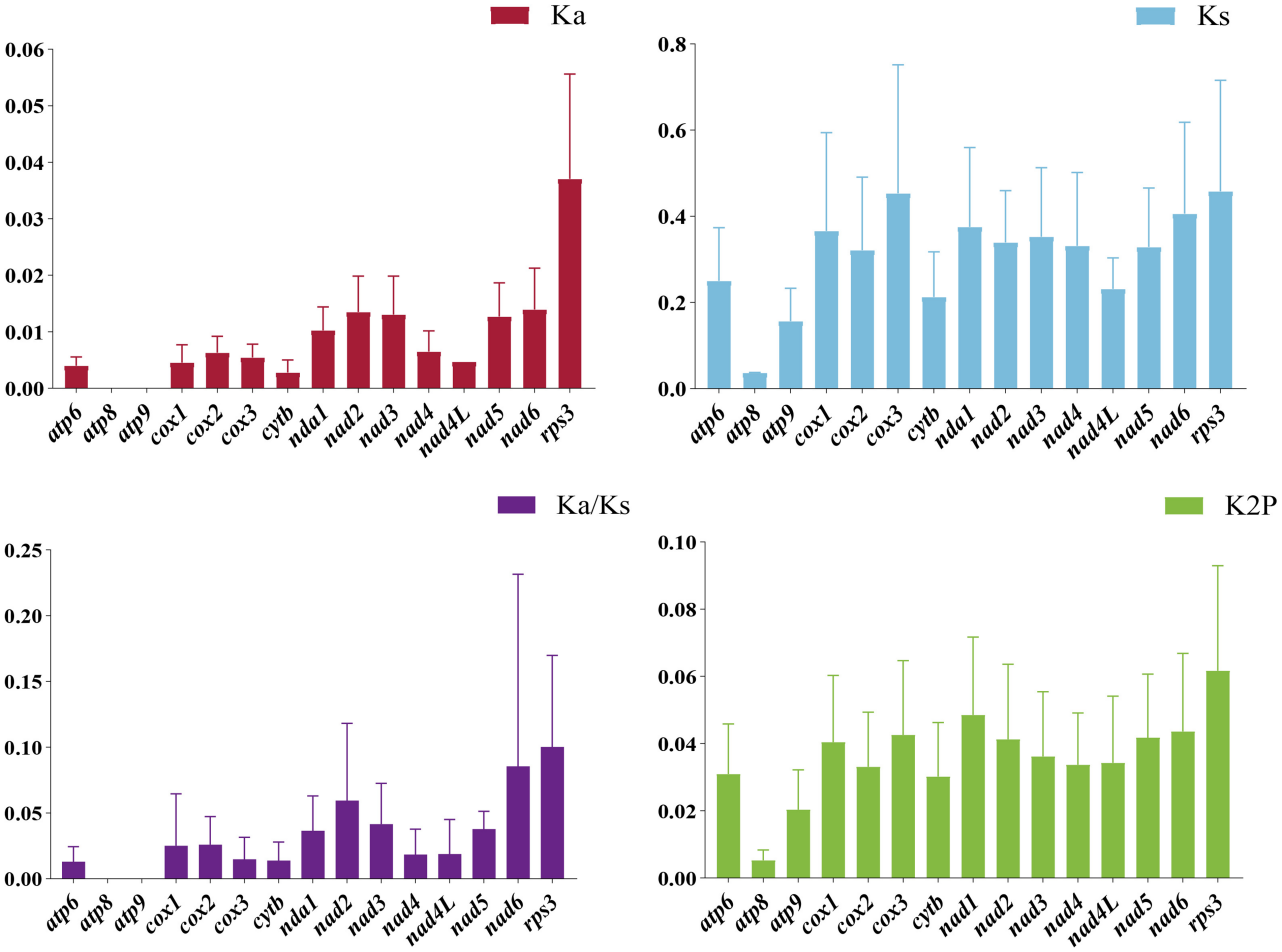
Genetic analysis of the 15 PCGs in the mitochondrial genomes of seven *Cordyceps* species. K2P: Overall mean of Kimura-2-Parameter distances; Ka: Mean number of nonsynonymous substitutions per nonsynonymous locus; Ks: Mean number of synonymous substitutions per synonymous locus.

Additionally, we examined the nonsynonymous substitution rate (Ka), synonymous substitution rate (Ks), and Kimura-2-parameter distance for the seven *Cordyceps* mitogenomes (Figure 6). Among the 15 PCGs, the *rps3* gene exhibited the highest genetic distance, indicating a higher mutation rate. Conversely, the *atp8* gene exhibited the lowest genetic distance, highlighting its high conservation. Furthermore, the *rps3* gene had the highest Ka/Ks ratio among the 15 PCGs, while the *atp8* and *atp9* genes had the lowest Ka/Ks values. All Ka/Ks values for the 15 PCGs were less than one, indicating that these genes were under purifying selection.

### Synteny analysis

**Figure 7:**
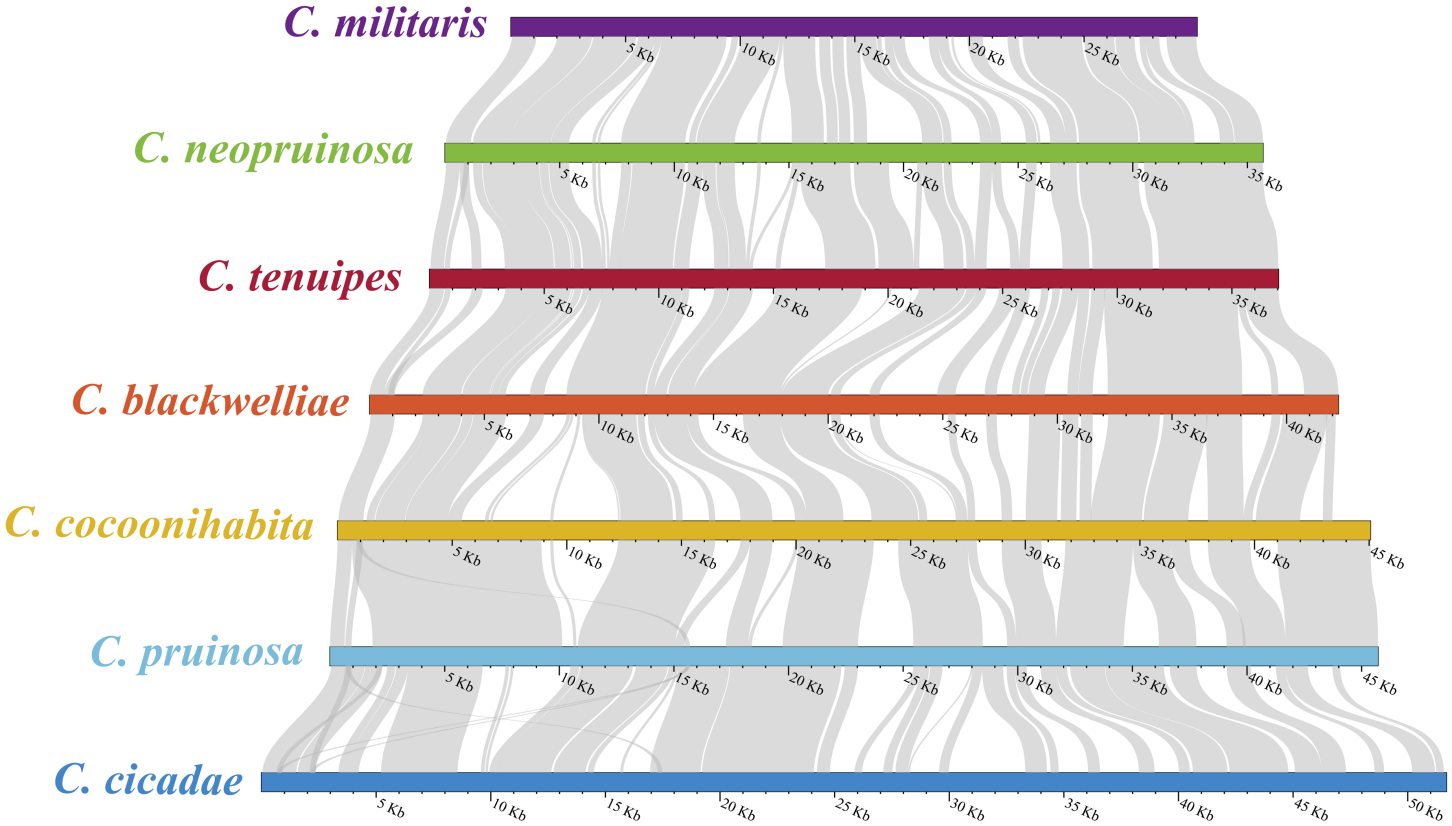
Covariance analysis of the complete mitogenomes of seven *Cordyceps* species. The gray-colored areas between species indicate varying degrees of homology.

The evolutionary relationships among the *Cordyceps* species were investigated through sequence comparison and covariance analysis (Figure 7). It was revealed that a considerable number of covariance regions existed in the seven species, with the majority exhibiting over 90% homology. The highest homology was found between *C. cicadae* and *C. pruinosa*, while the lowest was found between *C. pruinosa* and *C. cocoonihabita*. The longest segment, measuring approximately 4,884 bp, was found between *C. cicadae* and *C. pruinosa* among the homologous sequences, with a single mismatch of 24 bp.

### Phylogenetic analysis

**Figure 8:**
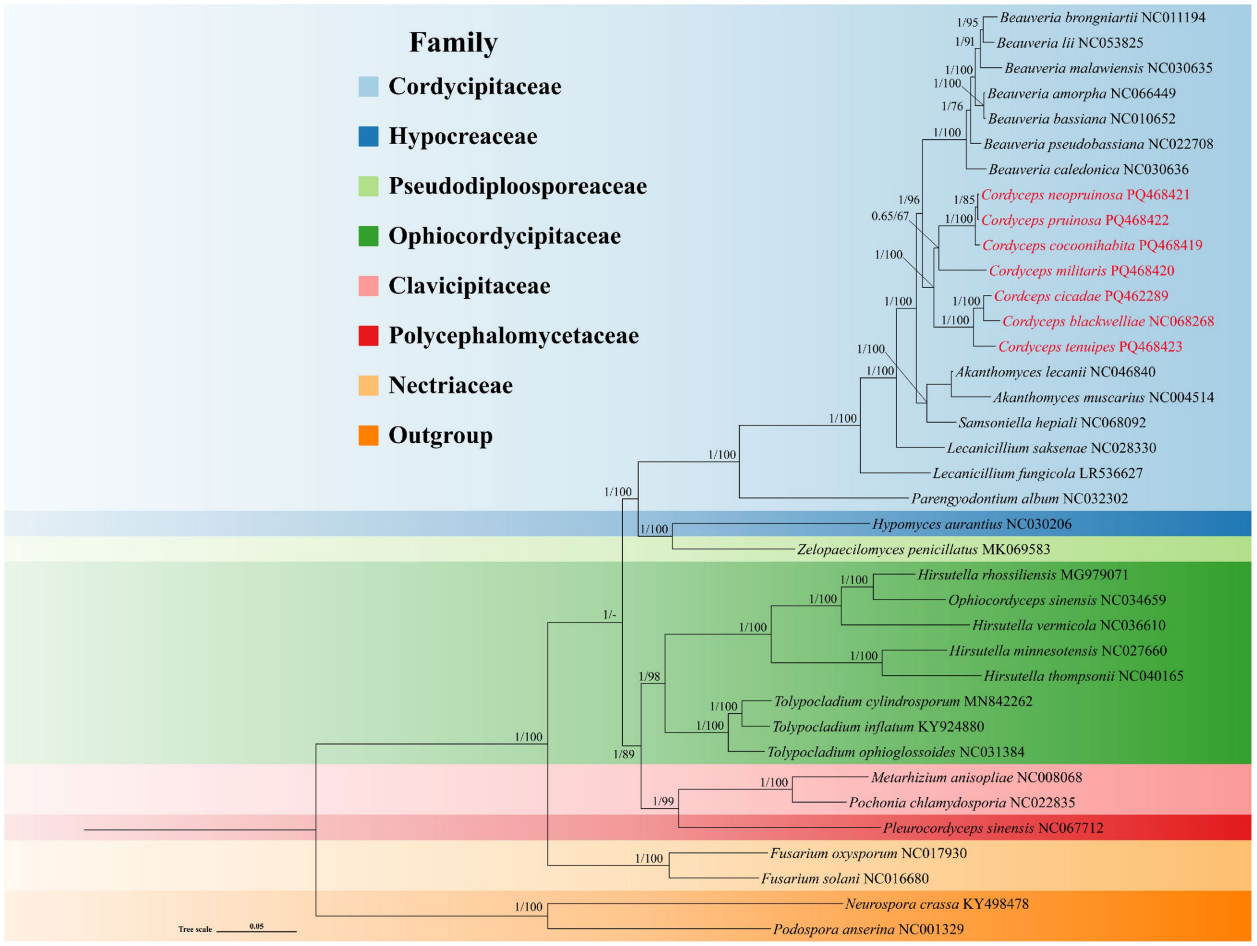
Phylogenetic relationships among 35 species of the order Hypocreales. The phylogenetic tree was constructed using 14 core PCGs through Bayesian inference (BI) and maximum likelihood (ML) methods, with *Neurospora crassa* and *Podospora anserina* designated as outgroups. The numbers at the nodes indicate Bayesian posterior probabilities (left) and bootstrap values (right). Detailed species names and accession numbers for the mitogenomes used in this phylogenetic analysis can be found in Supplementary Table S8.

A phylogenetic tree of the order Hypocreales was constructed using Bayesian Inference (BI) and Maximum Likelihood (ML) methods, based on 14 core PCGs (*atp6*, *atp8*, *atp9*, *cytb*, *cox1*, *cox2*, *cox3*, *nad1*, *nad2*, *nad3*, *nad4*, *nad4L*, *nad5*, and *nad6*). The resulting phylogenetic tree recognized strong support for all the distinct trunk cladaes (Figure 8). The 35 species of Hypocreales were placed in seven families: Cordycipitaceae, Hypocreaceae, Pseudodiploosporeaceae, Ophiocordycipitaceae, Clavicipitaceae, Polycephalomycetaceae, and Netriaceae. The seven *Cordyceps* species within the family Cordycipitaceae formed a monoplyletic clade, indicating a sister-relationship between *Cordyceps* and *Beauveria*. In particular, *C. cocoonihabita*, *C. neopruinosa*, *C. pruinosa*, and *C. militaris* closely clustered together in a subclade, while *C. blackwelliae*, *C. cicadae*, and *C. tenuipes* formed another distinct subclade.

## Discussion

### Variation in *Cordyceps* mitogenome size

In this study, we sequenced the mitogenomes of six *Cordyceps* species and performed comparative analysis with the previously published mitogenome of *C. blackwelliae*. Our analysis revealed significant size variation among the seven mitogenomes, with *C. cicadae* having the largest mitogenome (51,692 bp), while *C. militaris* exhibited the smallest (29,929 bp). This variability is consistent with patterns found in fungal mitogenomes, which are highly size-variable in eukaryotes due to dynamic intron changes, repetitive sequence accumulation, and non-conserved PCG variability (Al-Reedy et al., 2012; Mendoza et al., 2020; Deng et al., 2018; Wu et al., 2020). The largest mitogenome in *C. cicadae* contains 21 introns, which contribute a combined length of 23,347 bp, accounting for 45.16% of the entire genome. In contrast, the smallest mitogenome found in *C. militaris* contains only five introns, with a total length of 5,306 bp, accounting for 17.73% of the genome. The intron content plays a critical role in driving differences in mitogenome size among *Cordyceps* species. The evolutionary gain or loss of introns significantly contributes to the size variation in the genus *Cordyceps*. Interestingly, the mitogenome of *C. cocoonihabita* (45,058 bp) exhibits a 2,801 bp increase in size compared to that of *C. blackwelliae* (42,257 bp), despite containing 5,149 bp fewer intron nucleotides. This variation in size is likely attributed to the expansion or contraction of intergenic regions within the mitogenomes. Additionally, the expansion of non-conserved PCGs may further contribute to the overall increase in mitogenome size. The variation in mitogenome size among *Cordyceps* species is influenced by multiple factors, including but not limited to intron dynamics, changes in intergenic region length, and the accumulation of non-conserved PCGs.

### Evolution of the *Cordyceps* mitogenome content

Our results revealed a complete set of conserved core PCGs across all *Cordyceps* species. Minimal variations were found in the length, base composition, codon usage frequency, and start/stop codons of these PCGs, indicating that the core PCGs of *Cordyceps* have remained relatively conserved throughout evolution. However, the *rps3* gene displayed the highest Ka/Ks and K2P values among the 15 PCGs, indicating that it has undergone the most significant evolutionary changes. This phenomenon has also been reported in the mitochondria of other fungal species, though the selective pressures acting on *rps3* remain unclear (Wang Y. B. et al., 2020; Wang X. et al., 2020). Notably, the mitogenomes of *Cordyceps* species have varying numbers of non-conserved PCGs, many of which encode homing endonucleases and proteins of unknown function. The diversity of these non-conserved PCGs is remarkable. For example, the closely related species *C. blackwelliae*, *C. cicadae,* and *C. tenuipes* all contain *orf319*, while *C. cocoonihabita*, *C. neopruinosa*, and *C. pruinosa* harbor *orf293*, *orf299*, and *orf305*, respectively. These ORFs encode LAGLIDADG homing endonucleases. The recurrent gain and loss of these homing endonucleases and other non-conserved genes in *Cordyceps* species indicate/suggest an ongoing evolutionary fluctuation in the content of mitogenomes. The sizes of tRNA genes in the seven *Cordyceps* mitogenomes range from 71 bp to 85 bp. The variation in size is primarily influenced by the presence and size of extra arms in *trnL*, *trnS*, and *trnY*. It is well established that mutations in tRNA genes can impact protein synthesis and are linked to various diseases (Blakely et al., 2013; Yarham et al., 2010).

We identified three types of repetitive sequences in the *Cordyceps* mitogenomes: simple repeats, interspersed repeats, and tandem repeats. The analysis revealed significant variation in the distribution of these repetitive elements among *Cordyceps* species, predominantly located in the intergenic regions and introns. Repetitive sequences in fungal mitogenomes have been previously shown to be associated with genomic rearrangements (Ma et al., 2022). Our synteny analysis demonstrated high conservation in the PCGs regions of *Cordyceps* mitogenomes, while substantial differences were found in the non-coding regions. These findings suggest that repetitive sequences likely play a crucial role in driving the divergence found in the non-coding regions among *Cordyceps* species.

### Dynamics of introns in *Cordyceps* mitogenomes

Mitogenome size in fungi has been demonstrated to strongly correlate with the number of introns present among different lineages (Li et al., 2019). In this study, we detected a total of 84 introns within the mitogenomes of the seven *Cordyceps* species. The number of introns positively correlates with mitogenome size, indicating that introns are the primary contributors to mitogenome size variation. However, no significant correlation was found between the number of introns and the length of the corresponding host gene, indicating that specific sequence preferences rather than gene length determine the insertion sites for introns. Introns are unevenly distributed in PCGs and rRNA, with PCGs serving as the primary hosts. It is worth noting that the *cox1* gene harbors the highest number of introns among *Cordyceps* species. Introns can be classified into distinct Pcls based on their insertion sites, with homologous introns being assigned to identical Pcl designations (Li et al., 2020b). Among the seven *Cordyceps* species, the Pcls (P212, P721, and P1057) of the *cox1* gene were found to be the most prevalent. Throughout the evolutionary history of these species, intron loss has been noted, particularly in *C. tenuipes* and *C. militaris*, which only retain the P731 intron. In contrast, rare Pcls, such as P572 in the *atp6* gene, P25 and P823 in the *cob* gene, P51 and P218 in the *cox2* gene, P333, P219, and P631 in the *cox3* gene, P505 in the *nad4* gene, and P717 in the *nad5* gene, were detected in a single *Cordyceps* species. Interestingly, these rare Pcls have also been identified in distantly related species, suggesting the possibility of horizontal gene transfer events. Previous studies have similarly documented horizontal intron transfer between different organelles (Turmel et al., 1995). Both intron loss and gain events were found within *Cordyceps* mitogenomes, highlighting the dynamic nature of intron evolution in the genus *Cordyceps*. These findings indicate significant variation in the distribution of introns among *Cordyceps* species. However, further investigation is required to understand the functional implications and evolutionary dynamics of these introns.

### Molecular phylogeny

Mitogenomes have been extensively used in the phylogenetic analyses of animals, plants, and fungi (Jakovlić et al., 2023; Tang et al., 2023; Zhang et al., 2023; Zhong et al., 2022). The genus *Cordyceps*, widely distributed and species-rich within the family Cordycipitaceae (Panda and Swain, 2011), has been traditionally used as a medicinal tonic in Chinese medicine for thousands of years (Zhu et al., 1998). However, understanding the evolutionary relationships within *Cordyceps* has been challenging due to limited and overlapping morphological characteristics, as well as the inadequacies of multi-gene locus phylogenetics. Consequently, more robust tools that provide richer genetic information are needed for accurate phylogenetic analysis of *Cordyceps*. Mitogenomes have been widely used in population genetics and evolutionary research (Li et al., 2018). Because the independent evolution of mitogenomes and their large number of available molecular markers make mitogenomes the most attractive tool for analysing phylogenetic relationships between species (Jiang et al., 2017). Through comprehensive comparative analyses of mitochondrial genomes, our phylogenetic study strongly supports the close evolutionary relationships among the seven *Cordyceps* species. Phylogenetic tree for 35 species within the order Hypocreales was constructed using ML and BI methods, revealing high support rates for the major evolutionary branches. These results elucidate the phylogenetic relationships among *Cordyceps* species and clarify their phylogenetic positions within Hypocreales. This study provides valuable reference data for the classification and identification of *Cordyceps* species, contributing to a deeper understanding of interspecific variation within the genus. Additionally, the findings suggest phylogenetic relationships among *Cordyceps* species, offering genetic insights that could aid in the large-scale development and utilization of medicinally important *Cordyceps* species.

## Conclusion

In this study, the complete mitogenomes of six *Cordyceps* species were sequenced, assembled, and annotated. Through a comprehensive comparative analysis of seven *Cordyceps* mitogenomes, we revealed both conserved and variable features. The results demonstrate significant variation in mitogenome size among *Cordyceps* species, primarily driven by differences in intron numbers, with intron loss events occurring during evolution processes. Based on intronic Pcls, PCGs are the primary hosts, with Pcls of the *cox1* gene (P212, P721, and P1057) being the most prevalent. Additionally, various types of repeat sequences, including tandem repeats, dispersed repeats, and simple sequence repeats (SSRs), were predominantly detected within intergenic regions and introns. Among the core PCGs, *rps3* exhibited positive signs or relaxed selection, while the non-conserved PCGs showed dynamic variations among *Cordyceps* species. Phylogenetic analysis confirmed that *Cordyceps* species form a distinct and well-supported lineage within the family Cordycipitaceae, thus highlighting the reliability of mitochondrial PCGs in resolving the phylogenetic relationships within the geus *Cordyceps*. This study contributes to the existing mitogenome data for *Cordyceps* species and provides new insights into inter-specific variations, thereby enhancing our understanding of the evolutionary dynamics and genetic diversity within *Cordyceps*.

## Data Availability

The datasets presented in this study can be found in online repositories. The names of the repository/repositories and accession number(s) can be found at: https://www.ncbi.nlm.nih.gov/genbank/, PQ462289, PQ468419, PQ468420, PQ468421, PQ468422, and PQ468423.

## Footnotes

^1^ https://nmdc.cn/fungalnames/

^2^ https://www.ncbi.nlm.nih.gov/nuccore/?term=cordyceps
