# Supplementary material for: Mitochondrial genome characterization, evolution and intron dynamics of the entomopathogenic genus Cordyceps
Source: Front Microbiol. 2025 Jun 13;16:1605218. doi: 10.3389/fmicb.2025.1605218 (PMC12202336; doi:10.3389/fmicb.2025.1605218)
Supplement: Supplementary file 1 [file Data_Sheet_1.ZIP › Supplementary files/supplementary Figure.docx]

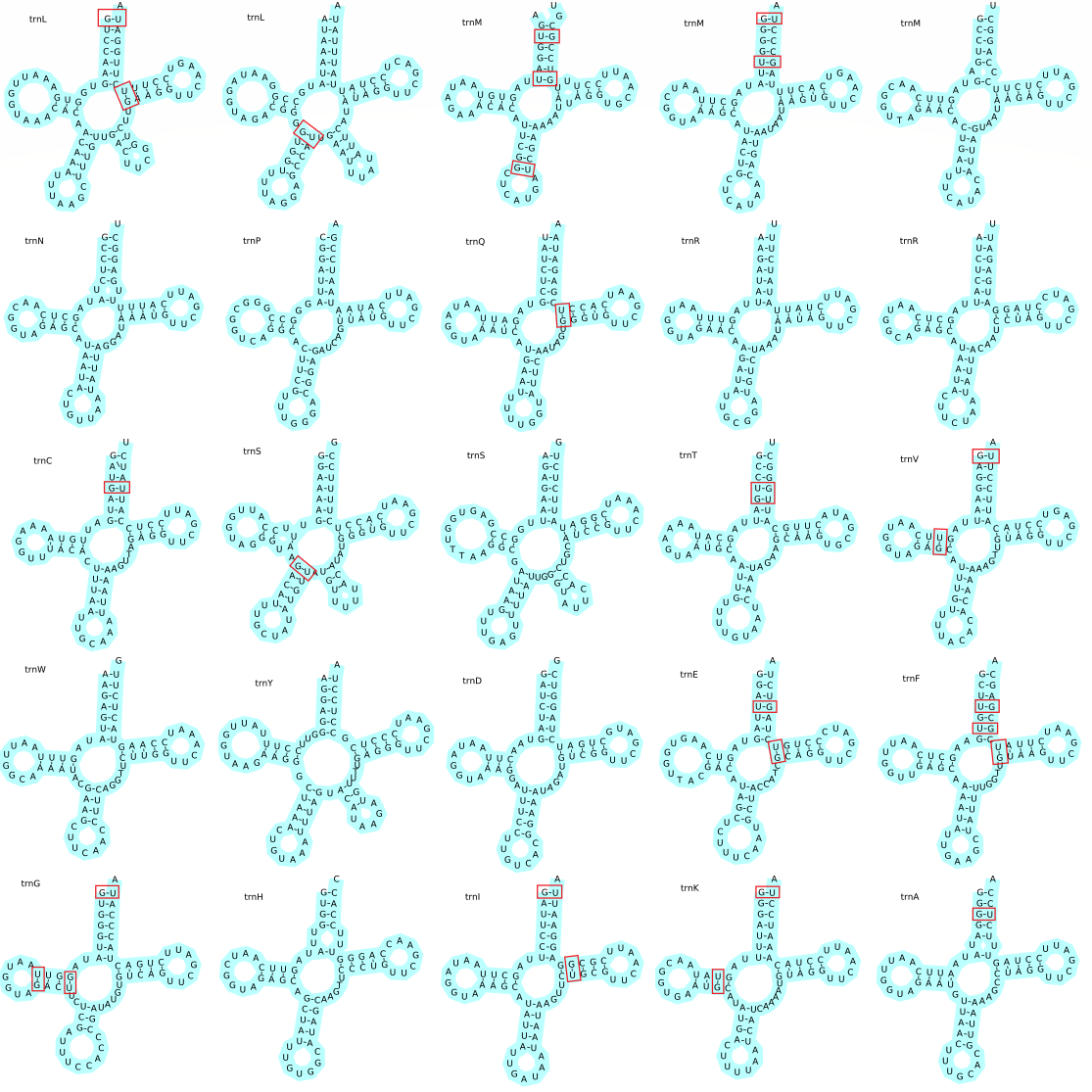


**Figure S1** The predicted secondary structures of the shared tRNAs of the mitochondrial genomes of the seven *Cordyceps* species are shown. G-U mismatch sites are labeled in the figure.


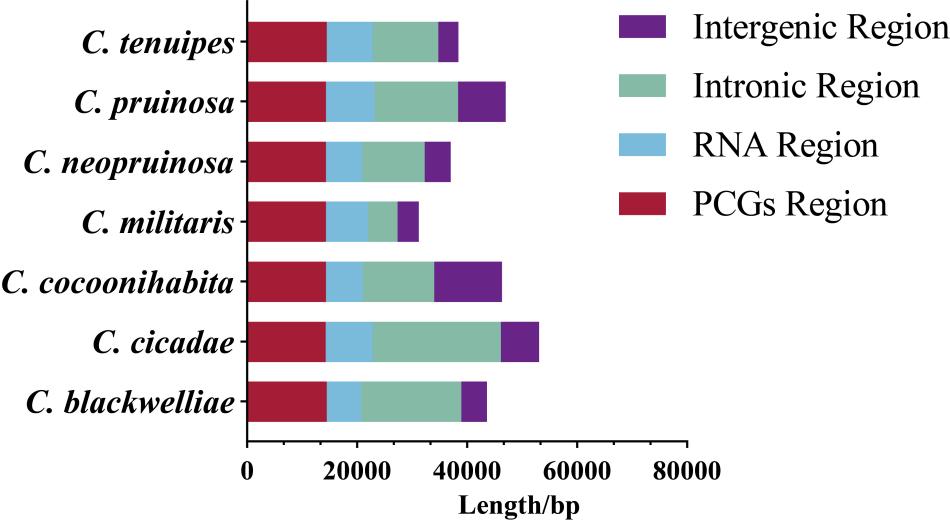


**Fiugre S2** Abundance of Intergenic Region, Intronic Region, RNA Region and PCGs Region regions in the mitochondrial genome of seven *Cordyceps* species.


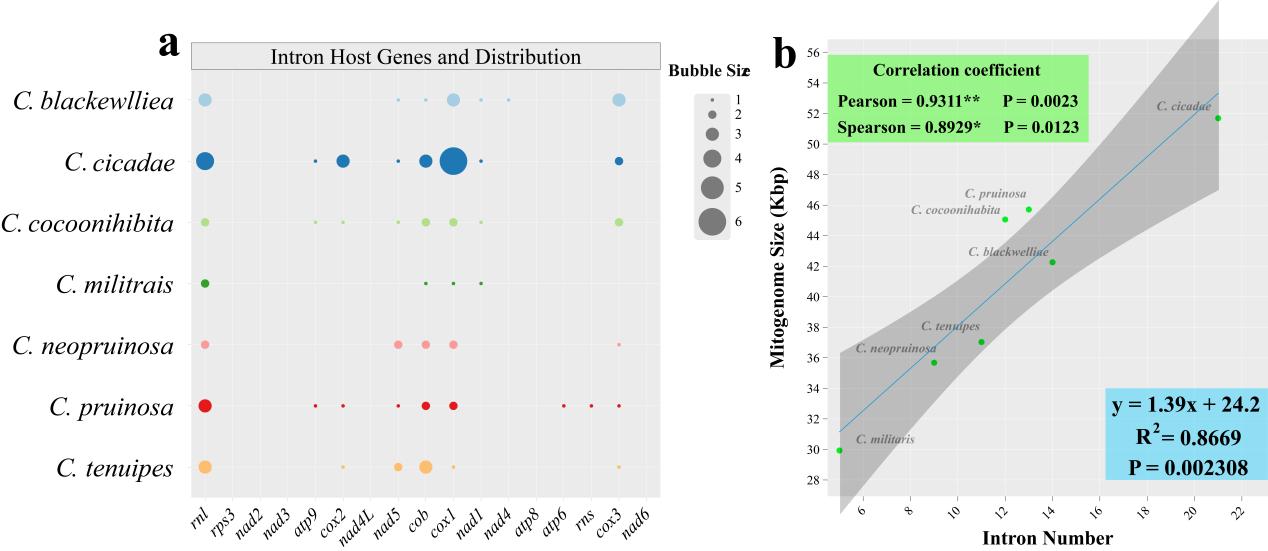


**Figure S3** (a) Intron host genes and distribution in the mitochondrial genomes of seven *Cordyceps* species (b) Correlation analysis between the number of introns in the mitochondrial genomes of *Cordyceps* species and the size of the mitochondrial genome.
