# Supplementary figures and images for: Mitochondrial genome characterization, evolution and intron dynamics of the entomopathogenic genus Cordyceps
Source: Front Microbiol. 2025 Jun 13;16:1605218. doi: 10.3389/fmicb.2025.1605218 (PMC12202336; doi:10.3389/fmicb.2025.1605218)

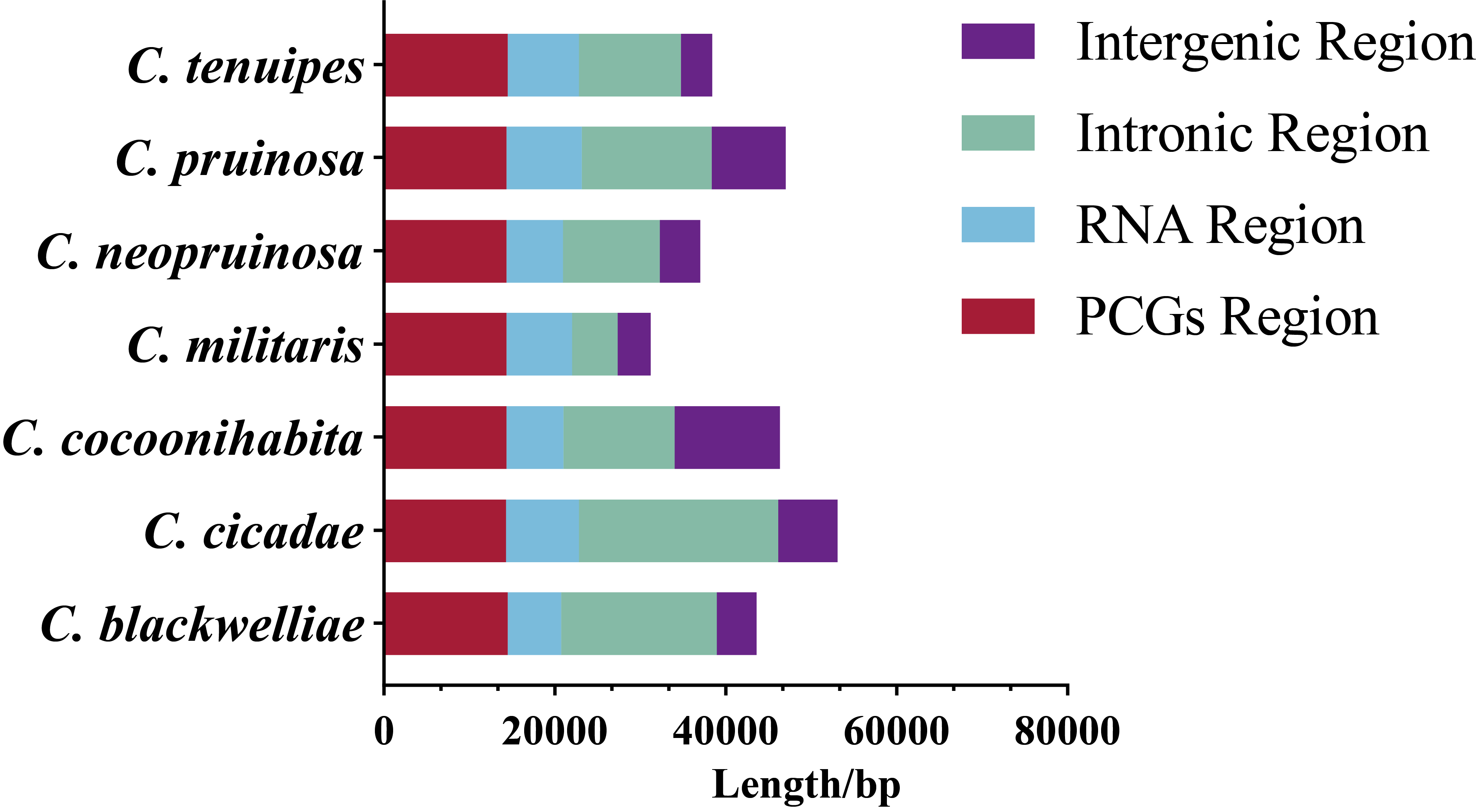

Supplement: Supplementary file 1 [file Data_Sheet_1.ZIP › Supplementary files/Figure S2.jpg]
